# Relationship Between the Early Initiation of Substance Use and Attempted Suicide Among in-School Adolescents in Seven Low- or Middle-Income African Countries: An Analysis of the Global School-Based Student Health Survey Data

**DOI:** 10.3389/fpsyg.2021.753824

**Published:** 2021-11-11

**Authors:** Lian Li, Yuanzhi Zhao, Meijun Shi, Yucheng Wang

**Affiliations:** Ningbo Kangning Hospital, Ningbo, China

**Keywords:** attempted suicide, early initiation of substance use, adolescents, Africa, LMICs

## Abstract

**Background:** Preventing suicide among adolescents is an urgent global public-health challenge, especially in Africa. Accordingly, the aim of this study was to examine the relationship between the early initiation (< 12 years old) of substance use (cigarette smoking, alcohol use, and drug use) and attempted suicide among in-school adolescents in seven African countries.

**Methods:** Data on the early initiation of substance use and on attempted suicide among in-school adolescents over the previous 12 months in Benin, Liberia, Mauritius, Mozambique, Namibia, Seychelles, and the United Republic of Tanzania were collected from Global School-based Student Health Surveys and were pooled to determine the overall prevalence of these behaviors in adolescents. Univariate and multivariate logistic regressions were then performed to evaluate country-specific associations between the early initiation of substance use and attempted suicide in these adolescents, followed by meta-analyses to evaluate overall pooled associations.

**Results:** In the abovementioned seven African low- or middle-income countries (LMICs), overall weighted prevalences of attempted suicide and early initiation of cigarette smoking, alcohol use, and drug use among in-school adolescents were 16.05, 7.76, 17.68, and 3.48%, respectively. Multivariate logistic regression analyses revealed that relative to non-smoking, the early initiation of smoking was significantly associated with attempted suicide in these adolescents [OR (95% CI) = 1.783 (1.219–2.348)]. Additionally, the relationship between early initiation of cigarette smoking and attempted suicide is mostly driven by a higher association in girls [OR (95% CI) = 1.867 (1.031–2.703)] than boys [OR (95% CI) = 1.392 (0.995−1.789)]. Moreover, relative to not using other drugs, the early and later initiation of other drug use were also significantly associated with attempted suicide in these adolescents [ORs (95% CIs) = 2.455 (1.701–3.208) and 1.548 (1.198–1.898)].

**Conclusion:** Programs that can eliminate or decrease the early initiation of substance use among adolescents should be implemented in African LMICs to prevent subsequent suicide attempts, especially among adolescent girls.

## Introduction

The prevention of adolescent suicide is an urgent global public-health challenge (Arensman et al., 2020). In 2016, more than 62,000 adolescents worldwide died by suicide, with more than 90% of these deaths occurring in low- and middle-income countries (LMICs) (World Health Organization, 2020). Suicide attempt is the strongest predictor of and risk factor for further suicide attempts and completed suicide (Swahn et al., 2012b; Bostwick et al., 2016). Thus, it is vital to clarify the factors associated with suicide attempts. This is in keeping with the United Nations Sustainable Development Goals (SDGs) Indicator 3.4.2, which emphasizes reducing the suicide mortality rate as one of the ways to reduce premature mortality from non-communicable diseases by one-third by 2030 (United Nation, 2020).

Africa has the world’s largest population of children, and the world’s second-largest population of adolescents (Almansour and Siziya, 2017). Approximately 17% of adolescents in Africa have self-reported having attempted suicide more than once in the previous 12 months, and this prevalence is higher than other LMICs regions (McKinnon et al., 2016; Li et al., 2021). It is therefore crucial to devise and implement suitable suicide-prevention programs in Africa to save lives and to meet the above SDG indicator.

Substance use, such as smoking cigarettes, drinking alcohol, and using drugs, is strongly associated with attempted and completed suicide (Agrawal et al., 2017; Michael and Saeed, 2018). Recently, there has been renewed interest in the pivotal role that the early initiation of substance use plays in suicidal behaviors, especially in adolescents. Findings from school-based health surveys in the United States (Swahn and Bossarte, 2007), South Korea (Kim and Kim, 2010), and four Pacific Island countries (Peltzer and Pengpid, 2015) have shown that, compared with those adolescents who did not use substances, early initiation of substance use in adolescents were more likely to report having attempted suicide. Moreover, an 8-year follow-up study in Mexico showed that adolescents who began using cannabis and alcohol at an early age had a 5.23 and 1.79 times greater risk of attempting suicide than those who did not (Borges et al., 2017). Furthermore, a study of a large population-representative sample of adult Australian twins revealed that relative to the absence of early initiation of alcohol use, the early initiation of alcohol use quadrupled the risk of subsequent attempted suicide (Few et al., 2015).

The reported associations between these dramatically increased risks of attempted suicide in adolescents and the early initiation of substance use are consistent and unambiguous, yet the specific mechanisms underlying these associations remain unclear. It may be that the early initiation of substance use by adolescents is due to a complex interaction between developmental, psychological, and sociocultural factors, which jointly increase the risk of attempted suicide (Bridge et al., 2006; Swahn and Bossarte, 2007). In addition, younger adolescents appear more vulnerable to adverse health outcomes from substance use, often resulting in chronic injuries, exposure to violence, and suicide (Swahn et al., 2012a).

However, little is known about the relationship between the early initiation of substance use and attempted suicide in Africa, which has the world’s highest rate of attempted suicide in adolescents (Li et al., 2021). Therefore, the aim of this study was to explore the prevalence of the early initiation of substance use (cigarette smoking, alcohol use, and other drug use) and attempted suicide among in-school adolescents in seven African countries (LMICs), and then examine the relationship between these behaviors.

## Materials and Methods

### Survey Details

The data used for this study were obtained from the publicly available datasets of the Global School-based Student Health Survey (GSHS), which is a self-administered, school-based multi-country survey that has been conducted in LMICs and was jointly developed by the World Health Organization (WHO) and the United States Centers for Diseases Control and Prevention (CDC) (Xi et al., 2016; Campisi et al., 2020). The GSHS uses a standardized two-stage probability sampling design for the selection process in each participating LMIC. In the first stage, probability-proportional-to-size sampling is used to randomly sample schools from all schools in each LMIC. In the second stage, systematic equal-probability sampling is used to randomly sample classes containing target-age students in each selected school. All students in the selected classes are eligible participants. All surveys were approved by the Ministry of Health or Education and Ethics Committee of each LMIC. We used the most recent survey datasets from LMICs that participated in more than two surveys, and we excluded survey datasets that lacked any variables that were relevant to our analysis. Ultimately, datasets from surveys performed in seven African LMICs from 2013 to 2017 were included in this study. The characteristics of each included LMIC are shown in Table 1.

**TABLE 1:** Survey characteristics by country among in-school African adolescent.

**Countries (Survey year)**	**N**	**Response rate (%)**	**Boys (%)**	**Suicide attempt (%)^a^**
Benin (2016)	1,563	79.38	55.47	14.20 (10.78-17.61)
Liberia (2017)	1,491	82.14	53.59	27.40 (23.13-31.67)
Mauritius (2017)	2,497	94.65	45.13	11.43 (9.28-13.58)
Mozambique (2015)	1,402	92.96	53.85	15.57 (11.73-19.41)
Namibia (2013)	3,183	89.78	46.78	20.87 (17.79-23.95)
Seychelles (2015)	2,013	89.13	43.47	16.13 (13.92-18.29)
United Republic of Tanzania (2014)	2,664	91.64	48.95	7.99 (6.91-9.08)
Overall	14,813	89.28	48.71	16.05 (11.47-20.64)

*^a^Weighted percentage of attempted suicide.*

### Measures

The data used for this study were collected via a self-reported questionnaire, which was translated into the local language of each LMIC and completed by students on a computer-scannable form during regular class time. The details of measures pertaining to this analysis are detailed in Supplementary Table 1. Specifically, the number of suicide attempts by adolescents in the previous 12 months were dichotomized as 0 times and 1 or more times. Cigarette-use initiation, alcohol-use initiation, and drug-use initiation were trichotomized as never, before 12 years of age (early), and at 12 years of age or older (later). Other potential confounders (insufficient food, having a physical fight, being bullied, current cigarette smoking, current alcohol use, current cannabis use, individual items regarding psychological distress, and individual items regarding parental support) were also dichotomized. Ultimately, psychological distress and parental support were calculated as the sum of individual items.

### Statistical Analysis

The weighted prevalence of attempted suicide and the early initiation of substance use were calculated using the SURVEYMEANS procedure in SAS software (version 9.4; SAS Institute, Cary, NC, United States). We added weights, strata, and a primary sampling unit to every set of school-attending adolescents to reflect the weighting process and the two-stage sampling design. We also used univariate logistic regression models to analyze the weighted relationships in each African LMIC between the early initiation of substance use, confounding factors, and attempted suicide, and then adjusted for confounding factors in multivariate logistic regression models to explore the relationships between the early initiation of substance use and attempted suicide. We calculated pooled overall estimates by fixed-/random-effects meta-analysis using STATA (version 12.0; Stata Corporation; College Station, TX, United States), and used the Higgins’s *I*^2^ statistic to estimate between-country heterogeneity. A between-country heterogeneity value of < 25% was considered to indicate negligible heterogeneity, and relevant data were thus subjected to a fixed-effects meta-analysis;(Higgins et al., 2003) whereas a heterogeneity value of ≥ 25% was considered to indicate positive heterogeneity, and relevant data were thus subjected to analysis using a random-effects model. *P*-values < 0.05 were considered as statistically significant. All statistical analyses were performed using SAS version 9.4 and STATA version 12.0.

## Results

**FIGURE 1:**
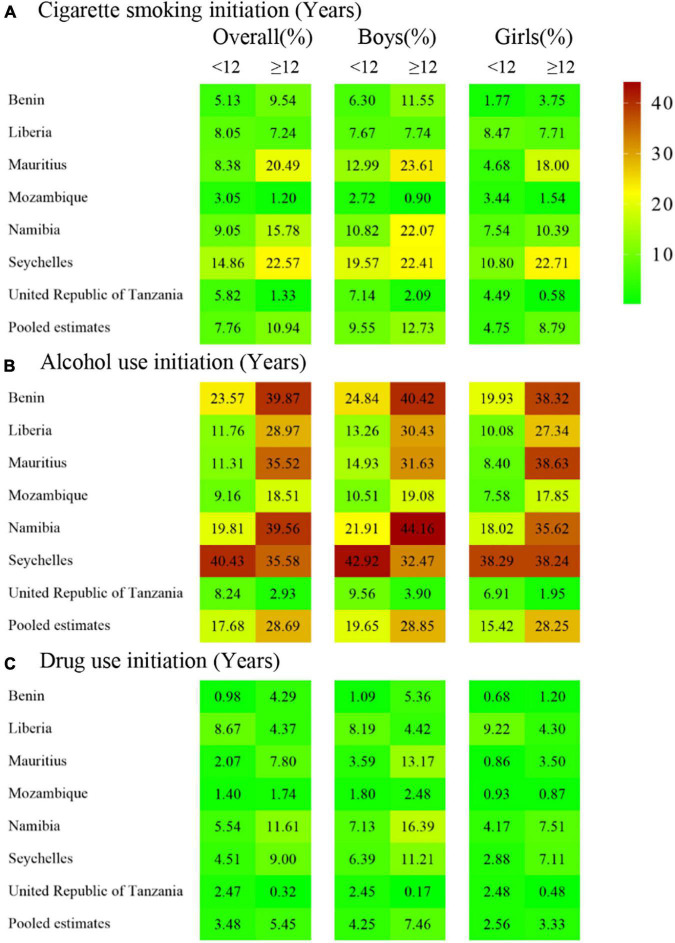
Prevalence (%) of the early and later initiation of substance use, namely **(A)** cigarette smoking, **(B)** alcohol use, and **(C)** drug use, by country and gender among in-school African adolescents.

Table 1 shows that the overall response rate was 89.28%, ranging from 79.38% in Benin to 94.65% in Mauritius. The final sample comprised 14,813 in-school adolescents (48.71% boys and 51.29% girls). The overall weighted prevalence of attempted suicide in the past 12 months was 16.05% (11.47–20.64), ranging from 7.99% (6.91–9.08) in the United Republic of Tanzania to 27.40% (23.13–31.67) in Liberia (Table 1). The overall weighted prevalence of attempted suicide in girls was 16.45%, and higher than 15.39% in boys (Supplementary Table 2). Moreover, the weighted prevalence of the early initiation of cigarette smoking (< 12 years) was 7.76% (9.55% in boys and 4.75% in girls), that of alcohol use was 17.68% (19.65% in boys and 15.42% in girls), and that of drug use was 3.48% (4.25% in boys and 2.56% in girls) (Figure 1 and Supplementary Tables 3–5). The weighted prevalence of the early initiation of substance use varied in countries, with all *I*^2^ were greater than 75% (Supplementary Tables 3–5).

**FIGURE 2:**
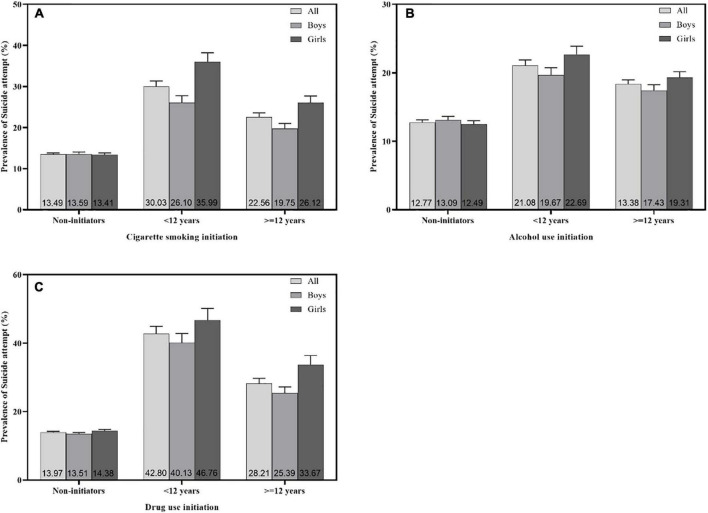
Prevalence (%) of attempted suicide by initiation of substance use, namely **(A)** cigarette smoking, **(B)** alcohol use, and **(C)** other drug use, among in-school African adolescents. Bars denote standard error.

The prevalence of attempted suicide in adolescents who had early and later initiators of cigarette smoking in the previous 30 days (30.03 and 22.56%) were higher than those who had not smoked (13.49%, Figure 2). The prevalence of attempted suicide in adolescents who had early and later initiators of alcohol use in the previous 30 days (21.08 and 13.38%) were higher than those who had not used alcohol (12.77%, Figure 2). The prevalence of attempted suicide in adolescents who had early and later initiators of other drug use in the previous 30 days (42.80 and 28.21%) were higher than those who had not used drugs in the previous 30 days (13.97%, Figure 2). Adolescent girls who were early or later initiators of substance use had higher prevalences of attempted suicide than the corresponding groups of boys (Figure 2).

The early initiation of cigarette smoking, alcohol use, and other drug use (< 12 and ≥ 12 years old) were all significantly associated with attempted suicide in adolescents. Current cigarette smoking, alcohol use, drug use, insufficient food, having a physical fight, and being bullied were also significantly associated with attempted suicide. Furthermore, psychological distress and parental support were also significantly associated with attempted suicide, and the magnitude of these associations increased with the number of items (Table 2).

**TABLE 2:** Unadjusted relationships between study variables and attempted suicide among in-school African adolescent.

	**Unadjusted odds ratios**	***P*-value**	**I^2^**
Experience hungry	1.964 (1.630-2.298)	<0.001	18.0%
Having a physical fight	2.106 (1.639-2.572)	<0.001	69.0%
Be bullied	2.521 (2.049-2.993)	<0.001	52.9%
**Number of psychological distress (ref = 0)**			
1	1.809 (1.415-2.202)	<0.001	56.2%
2	3.228 (1.884-4.573)	<0.001	60.5%
**Number of parental support (ref = 0)**			
1	0.701 (0.488-0.913)	<0.001	73.0%
2	0.542 (0.342-0.743)	<0.001	77.8%
3	0.398 (0.241-0.554)	<0.001	60.0%
Current cigarette smoking	2.915 (1.878-3.952)	<0.001	68.7%
Current alcohol use	1.560 (1.159-1.961)	<0.001	70.2%
Current drug use	3.614 (1.991-5.237)	<0.001	54.0%
**Initiation of cigarette smoking (ref = Non-initiators)**			
< 12 years	5.582 (1.718-3.447)	<0.001	70.1%
≥ 12 years	1.913 (1.232-2.593)	<0.001	68.7%
**Initiation of alcohol use (ref = Non-initiators)**			
< 12 years	1.721 (1.212-2.229)	<0.001	70.5%
≥ 12 years	1.301 (1.014-1.587)	<0.001	46.6%
**Initiation of drug use (ref = Non-initiators)**			
< 12 years	4.164 (2.550-5.777)	<0.001	52.3%
≥ 12 years	2.054 (1.496-2.612)	<0.001	30.9%

Multivariate logistic regression analyses revealed that relative to non-smokers, the early initiation of cigarette smoking in adolescents was significantly associated with attempted suicide [OR (95% CI) = 1.783 (1.219–2.348)]. However, the early initiation of alcohol use was not significantly associated with attempted suicide [OR (95% CI) = 1.168 (0.832–1.503)]. The early and later initiation of drug use were also significantly associated with attempted suicide [ORs (95% CIs) = 2.455 (1.701–3.208) and 1.548 (1.198–1.898), respectively], and there was a significant association between the early initiation of drug use and attempted suicide in both adolescent girls and boys [ORs (95% CIs) = 2.446 (1.299–3.593) and 2.094 (1.258–2.931)]. Additionally, the relationship between early initiation of cigarette smoking and attempted suicide is mostly driven by a higher association in girls [OR (95% CI) = 1.867 (1.031–2.703)] than boys [OR (95% CI) = 1.392 (0.995−1.789)] (Table 3).

**TABLE 3:** Adjusted^a^ relationships between the early initiation of substance use and attempted suicide among in-school African adolescent.

	**Total**		**Boys**		**Girls**	
	**Adjusted odds ratios**	**I^2^**	**Adjusted odds ratios**	**I^2^**	**Adjusted odds ratios**	**I^2^**
**Cigarette smoking initiation (ref = Non-initiators)**						
< 12 years	1.783 (1.219-2.348)^*^	55.5%	1.392 (0.995-1.789)^#^	10.2%	1.867 (1.031-2.703)^*^	27.6%
≥12 years	1.498 (0.960-2.036)	56.1%	1.155 (0.818-1.492)^#^	0.0%	1.482 (0.636-2.327)	61.5%
**Alcohol use initiation (ref = Non-initiators)**						
<12 years	1.168 (0.832-1.503)	51.0%	0.875 (0.661-1.089)	4.5%	1.288 (0.796-1.780)	49.4%
≥12 years	1.051 (0.791-1.310)	40.6%	0.793 (0.620-0.966)^*^^#^	0.0%	1.088 (0.835-1.341)^#^	0.0%
**Drug use initiation (ref = Non-initiators)**						
<12 years	2.455 (1.701-3.208)^*^^#^	0.0%	2.094 (1.258-2.931)^*^^#^	0.0%	2.446 (1.299-3.593)^*^^#^	0.0%
≥12 years	1.548 (1.198-1.898)^*^	10.7%	1.509 (0.996-2.023)^#^	0.0%	1.279 (0.762-1.796)	9.7%

*^a^Adjusted for age, sex; insufficient food, having a physical fight; being bullied, psychological distress; parental support.*

*P < 0.001.

*^#^Pooled adjusted associations calculated by fixed-effects.*

## Discussion

Our findings demonstrated robust associations between the early initiation of cigarette smoking and drug use and self-reported attempted suicide in adolescents in Africa. The OR of self-reported attempted suicide in adolescents who were early initiators of cigarette smoking was 1.783 times that of adolescents who did not smoke. The relationship between early initiation of cigarette smoking and attempted suicide is mostly driven by a higher association in girls than boys. The OR of self-reported attempted suicide in adolescents who were early initiators of other drug use was 2.455 times that in those who did not use other drugs. However, the early initiation of alcohol use was not associated with self-reported attempted suicide.

The prevalences of the early initiation of cigarette smoking and other drug use in African adolescents were 7.8 and 3.5%, respectively, which was less than the prevalences that have been reported in previous studies of adolescents in Pacific Island countries (15.7 and 12.9%) (Peltzer and Pengpid, 2015), South-East Asian nations (10.6 and 4.2%) (Pengpid and Peltzer, 2019), France (24.1 and 3.9%), and the United States (18.1 and 9.7%) (Swahn et al., 2012a). In addition, the prevalence of the early initiation of alcohol use in African adolescents was 17.7%, which was much less than that in France (65.1%) and the United States (27.8%) (Swahn et al., 2012a), but greater than that in Pacific Island countries (13.8%) (Peltzer and Pengpid, 2015) and South-East Asian nations (8.1%) (Pengpid and Peltzer, 2019). Furthermore, we found that adolescents who were early initiators of substance use had higher rates of self-reported attempted suicide than those who were later initiators of substance use or not substance users. This demonstrates the great damage caused by the early initiation of substance use to African adolescents’ health and chances of surviving until adulthood and underscores the urgent need to decrease and ultimately prevent the early use of substances by these adolescents.

In our univariate analysis, experience hungry, having a physical fight, be bullied, psychological distress, parental support, current substance use were all associated with self-reported attempted suicide, which were contrast to previous studies (Li et al., 2021), and then we used multivariate logistic regression analysis to explore the independent relationship between the early initiation of substance use and attempted suicide. In line with previous studies, we found that the early initiation of cigarette smoking and drug use were positively associated with self-reported attempted suicide (Kim and Kim, 2010; Peltzer and Pengpid, 2015). It may be that the early initiation of cigarette smoking and drug use by adolescents is due to a complex interaction between developmental, psychological, and sociocultural factors, which jointly increase the risk of attempted suicide (Bridge et al., 2006; Swahn and Bossarte, 2007). However, in contrast to previous studies conducted in other LMICs (Peltzer and Pengpid, 2015; Pengpid and Peltzer, 2019) and developed countries (Cho et al., 2007; Swahn and Bossarte, 2007; Kim and Kim, 2010), after adjusting for confounding factors, we did not find a significant association between the early initiation of alcohol use and self-reported attempted suicide. This is consistent with the results from the 2003 Youth Risk Behavior Survey in the United States, which showed by multivariate analysis that there was no association between the early initiation of alcohol use and attempted suicide in adolescents (Swahn et al., 2012a).

The reasons for the above-mentioned differences between our findings and those of previous studies remain unclear, but there are several reasons that may account for the lack of an association between the early initiation of alcohol use and attempted suicide. First, although the early initiation of substance use typically harms the developing brains of adolescents (Guerri and Pascual, 2019), it was also found that low to moderate alcohol use may be associated with better global cognition scores in middle-aged to older adults (Zhang et al., 2020). Moreover, there is no safe level of exposure to cigarette smoke or other drugs: (World Health Organization, 2020) their ingestion is invariably harmful to the body. However, the paucity of data on the effects of various levels of substance use meant that we were unable to perform dose–response analyses. Second, the lack of association might be related to different normative use of alcohol in adolescents in developing and developed countries. Final, previous studies have verified that the association between the early initiation of substance use and attempted suicide is mediated by the effects of other factors (e.g., psychological distress or a lack of parental support) (Boden et al., 2008; Rossow et al., 2009; Hockenberry et al., 2010). Thus, we adjusted for psychological distress and parental support to determine whether there was a direct relationship between the initiation of substance use and attempted suicide in adolescents. After adjusting for confounding factors, the results revealed that the early initiation of cigarette smoking and drug use were directly related to attempted suicide, which suggests that these are key factors contributing to attempted suicide in adolescents.

In contrast, we found that the early initiation of cigarette smoking was not associated with attempted suicide in adolescent boys, which was consistent with the results from studies in South Korea (Kim and Kim, 2010), and in France and the United States (Swahn et al., 2012a), i.e., the early initiation of cigarette smoking was only associated with attempted suicide in adolescent girls. Although the reasons for this stark sex-based variation are unclear, they may be related to girls’ greater susceptibility to the effects of nicotine (Cross et al., 2017), which could result in sex-specific biological effects of nicotine on brain development. Therefore, future research should be conducted to explore the underlying mechanisms of the sex-related variation in the association between the early initiation of cigarette smoking and attempted suicide in adolescents, and to develop measures targeted to adolescent girls to prevent them from initiating early cigarette smoking and thus decrease their risk of subsequent suicide attempts.

## Strengths and Limitations

In our previous study, we highlighted the extremely high prevalence of suicidal behaviors in African adolescents (Li et al., 2021), and the current study extends this work by performing the first examination of the link between the early initiation of substance use and attempted suicide in African adolescents. We adjusted for numerous potential confounders (e.g., inadequate food, having a physical fight, being bullied, psychological distress, and a lack of parental support) that might account for indirect associations between the early initiation of substance use and attempted suicide. In addition, all data were processed by domain analyses, as GSHS has a complex sampling design consisting of strata, clusters, and weights. Furthermore, the associations were based on pooled data from all seven African countries surveyed and were determined by meta-analysis to account for the differences between countries.

However, there are several limitations to our study. First, the data from the GSHS are obtained via self-report, and thus, recall bias was inevitable. However, the GSHS questionnaire was validated by expert consultation (Xi et al., 2016), and thus, the self-reported nature of the questionnaire may not have had an adverse effect on our findings. Second, inadequate food was used as a surrogate for socioeconomic status, and if this was an incomplete surrogate, residual confounding of socioeconomic status may have been present. Third, the adolescents recruited for this study were all in school, but many African adolescents are not in school due to poverty or societal unrest. Thus, the conclusions in our study are relevant only for in-school adolescents. Fourth, only seven countries in Africa were included in our study, as 10 countries were excluded due to a lack of data. Finally, causal inferences between the early initiation of substance use and attempted suicide could not be determined due to the cross-sectional study design of the GSHS.

## Conclusion

We found that there was a high rate of early initiation of substance use (cigarette smoking, alcohol use, and drug use) and a high prevalence of attempted suicide among in-school adolescents in seven LMICs in Africa, and that the early initiation of cigarette smoking and drug use were associated with attempted suicide. Thus, it is critical that programs be developed to reduce or prevent the early initiation of substance use among adolescents in African, as this will decrease the possibility of them attempting suicide.

## Data Availability Statement

Publicly available datasets were analyzed in this study. This data can be found here: www.who.int/ncds/surveillance/gshs/en/.

## Author Contributions

LL, YZ, MS, and YW substantially contributed to the conception and the design of the work. YZ and MS participated in the acquisition of data. LL analyzed, interpreted the data, and prepared the draft. YW revised the manuscript. All authors worked for the final approval of the version to be published and were accountable for all the aspects of the work in ensuring that questions related to the accuracy and integrity of any part of the work are appropriately investigated and resolved.

## Conflict of Interest

The authors declare that the research was conducted in the absence of any commercial or financial relationships that could be construed as a potential conflict of interest.

## Publisher’s Note

All claims expressed in this article are solely those of the authors and do not necessarily represent those of their affiliated organizations, or those of the publisher, the editors and the reviewers. Any product that may be evaluated in this article, or claim that may be made by its manufacturer, is not guaranteed or endorsed by the publisher.
