# Supplementary material for: Relationship Between the Early Initiation of Substance Use and Attempted Suicide Among in-School Adolescents in Seven Low- or Middle-Income African Countries: An Analysis of the Global School-Based Student Health Survey Data
Source: Front Psychol. 2021 Nov 11;12:753824. doi: 10.3389/fpsyg.2021.753824 (PMC8631758; doi:10.3389/fpsyg.2021.753824)
Supplement: Supplementary file 1 [file Table_1.docx]

**Supplementary Table S1** Study measures, questions, and responses of study variables.

| **Variables** | **Question** | **Response and code** |
| --- | --- | --- |
| Age | How old are you? | 1=11 years old or younger, 2=12 years old, to 6=16 years old or older |
| Gender | What is your sex? | 1= male, 2= female |
| Insufficient food | During the past 30 days, how often did you go to hungry brcause there was not enough food in your home? | 0=never, rarely, or sometimes; 1=most of time or always |
| Having a physical fight | During the past 12 months, how many times were you in a physical fight? | 0= 0 times; 1=1 or more times |
| Being bullied | During the past 30 days, on how many days were you bullied? | 0= 0 times; 1=1 or more times |
| Psychological distress | During the past 12 months, how often have you felt lonely? | 0=never, rarely, or sometimes; 1=most of time or always |
|  | During the past 12 months, how often have you been so worried about something that you could not sleep at night? |  |
| Parental support | During the past 30 days, how often did your parents or guardians check to see if your homework was done? |  |
|  | During the past 30 days, how often did your parents or guardians understand your problems and worries? |  |
|  | During the past 30 days, how often did your parents or guardians really know what you were doing with your free time? |  |
| Current smoking cigarettes | During the past 30 days, on how many days did you smoke cigarettes? | 0= 0 days; 1=1 or more days |
| Current alcohol use | During the past 30 days, on how many days did you have at least one drink containing alcohol? |  |
| Current drug use | During the past 30 days, how many times have you used marijuana? | 0= 0 times; 1=1 or more times |
| Early cigarette use initiation | How old were you when you first tried a cigarette? | 1 = I have never; 2 = younger than 12 years old ; 3 = 12 years old or older |
| Early alcohol use initiation | How old were you when you had your first drink of alcohol other than a few sips? |  |
| Early drug use initiation | How old were you when you first used drugs? |  |
| Suicide attempts | During the past 12 months, how many times did you actually attempt suicide? | 0= 0 times; 1=1 or more times |

**Supplementary Table S2** Prevalence (%) of suicide attempt by country and gneder among in-school African adolescents.

|  | Boys | | Girls | |
| --- | --- | --- | --- | --- |
| Countries (Survey year) | N | Suicide attempt (%) | N | Suicide attempt (%) |
| Benin (2016) | 867 | 14.30 (10.94-17.65) | 696 | 13.90 (10.54-17.27) |
| Liberia (2017) | 799 | 27.64 (22.70-32.58) | 692 | 27.13 (22.47-31.80) |
| Mauritius (2017) | 1127 | 9.00 (7.41-10.59) | 1370 | 13.38 (10.28-16.47) |
| Mozambique (2015) | 755 | 15.06 (11.47-18.65) | 647 | 16.17 (9.76-22.57) |
| Namibia (2013) | 1489 | 22.43 (18.71-26.14) | 1694 | 19.53 (16.45-22.60) |
| Seychelles (2015) | 875 | 14.72 (11.96-17.48) | 1138 | 17.35 (14.55-20.14) |
| United Republic of Tanzania (2014) | 1304 | 7.10 (5.66-8.54) | 1360 | 8.89 (7.24-10.55) |
| Overall | 7216 | 15.39 (10.94-19.85) | 7597 | 16.45 (12.04-20.85) |

**Supplementary Table S3** Prevalence (%) of the early initiation of substance use by country among in-school African adolescents.

|  | Cigarette smoking initiation | | | Alcohol use initiation | | | Drug use initiation | | |
| --- | --- | --- | --- | --- | --- | --- | --- | --- | --- |
| **Countries** | Non-initiators | <12 years | ≥12 years | Non-initiators | <12 years | ≥12 years | Non-initiators | <12 years | ≥12 years |
| Benin | 85.33  (79.70-90.96) | 5.13  (3.44-6.82) | 9.54  (5.16-13.91) | 36.55  (32.22-40.89) | 23.57  (19.34-27.80) | 39.87  (36.39-43.36) | 94.73  (91.97-97.49) | 0.98  (0.30-1.67) | 4.29  (1.76-6.82) |
| Liberia | 84.23  (80.33-88.12) | 8.05  (5.74-10.35) | 7.24  (5.61-9.84) | 59.27  (55.37-63.16) | 11.76  (10.16-13.36) | 28.97  (25.63-32.31) | 86.96  (83.95-89.96) | 8.67  (6.35-11.01) | 4.37  (3.14-5.58) |
| Mauritius | 71.13  (66.82-75.44) | 8.38  (6.66-10.09) | 20.49  (16.63-24.35) | 53.18  (48.52-57.83) | 11.31  (9.11-13.50) | 35.52  (31.39-39.64) | 90.13  (87.39-92.87) | 2.07  (0.84-3.30) | 7.80  (5.57-10.03) |
| Mozambique | 95.75  (93.52-97.98) | 3.05  (0.96-5.14) | 1.20  (0.28-2.12) | 72.32  (66.30-78.35) | 9.16  (5.94-12.39) | 18.51  (14.02-23.01) | 96.87  (95.71-98.02) | 1.40  (0.68-2.12) | 1.74  (0.91-2.57) |
| Namibia | 75.17  (71.58-78.76) | 9.05  (7.16-10.95) | 15.78  (13.34-18.21) | 40.63  (36.26-45.00) | 19.81  (16.51-23.11) | 39.56  (36.50-42.62) | 82.86  (80.10-85.61) | 5.54  (4.59-6.48) | 11.61  (9.20-14.02) |
| Seychelles | 62.57  (59.08-66.06) | 14.86  (13.09-16.63) | 22.57  (19.13-26.01) | 24.00  (20.81-27.19) | 40.43  (37.41-43.46) | 35.58  (31.35-39.78) | 86.49  (84.18-88.79) | 4.51  (3.24-5.77) | 9.00  (7.06-10.95) |
| United Republic of Tanzania | 92.85  (91.56-94.15) | 5.82  (4.79-6.84) | 1.33  (0.71-1.95) | 88.84  (86.85-90.82) | 8.24  (6.98-9.50) | 2.93  (1.81-4.04) | 97.21  (96.25-98.17) | 2.47  (1.62-3.32) | 0.32  (0.07-0.57) |
| Overall | 81.06  (72.00-90.12) | 7.76  (5.09-10.44) | 10.94  (6.22-45.66) | 53.54  (31.76-75.32) | 17.68  (10.53-24.83) | 28.69  (13.39-43.98) | 90.85  (86.88-94.83) | 3.48  (1.94-5.03) | 5.45  (2.83-8.08) |
| I^2^ | 98.6% | 94.3% | 98.4% | 99.6% | 98.6% | 99.5% | 96.9% | 94.3% | 97.5% |

**Supplementary Table S4** Prevalence (%) of the early initiation of substance use by country among in-school African adolescent boys.

|  | Cigarette smoking initiation | | | Alcohol use initiation | | | Drug use initiation | | |
| --- | --- | --- | --- | --- | --- | --- | --- | --- | --- |
| **Countries** | Non-initiators | <12 years | ≥12 years | Non-initiators | <12 years | ≥12 years | Non-initiators | <12 years | ≥12 years |
| Benin | 82.15  (74.97-89.32) | 6.29  (4.01-8.58) | 11.55  (6.01-17.10) | 34.74  (30.28-39.20) | 24.84  (20.47-29.20) | 40.41  (36.31-44.51) | 93.54  (89.83-97.26) | 1.08  (0.14-2.02) | 5.36  (1.96-8.75) |
| Liberia | 84.59  (80.95-88.23) | 7.66  (5.23-10.09) | 7.74  (5.61-9.86) | 56.30  (50.16-62.45) | 13.26  (10.13-16.39) | 30.42  (25.97-34.88) | 87.38  (84.43-90.34) | 8.19  (5.71-10.66) | 4.41  (2.73-6.10) |
| Mauritius | 63.40  (59.50-67.30) | 12.98  (11.33-14.64) | 23.60  (20.07-27.13) | 53.43  (49.38-57.48) | 14.93  (11.98-17.88) | 31.62  (27.49-35.76) | 83.24  (81.04-85.43) | 3.58  (1.58-5.58) | 13.17  (10.60-15.73) |
| Mozambique | 96.37  (93.70-99.05) | 2.72  (0.23-5.20) | 0.90  (0.267-1.54) | 70.40  (63.71-77.08) | 10.51  (5.99-15.03) | 19.08  (14.63-23.52) | 95.72  (93.65-97.78) | 1.80  (0.49-3.10) | 2.47  (1.40-3.54) |
| Namibia | 67.11  (63.26-70.96) | 10.82  (8.13-13.51) | 22.06  (19.08-25.04) | 33.93  (28.85-39.01) | 21.90  (17.68-26.12) | 44.15  (40.83-47.48) | 76.47  (72.89-80.06) | 7.12  (5.58-8.67) | 16.39  (13.52-19.25) |
| Seychelles | 58.02  (53.53-62.50) | 19.56  (16.61-22.52) | 22.40  (18.33-26.48) | 24.61  (20.69-28.52) | 42.91  (38.92-46.90) | 32.47  (27.85-37.09) | 82.40  (78.93-85.87) | 6.389  (4.42-8.35) | 11.20  (8.36-14.04) |
| United Republic of Tanzania | 90.76  (88.91-92.61) | 7.14  (5.81-8.47) | 2.08  (0.97-3.19) | 86.54  (83.87-89.21) | 9.55  (7.53-11.57) | 3.89  (2.34-5.44) | 97.38  (96.22-98.54) | 2.45(1.34-3.56) | 0.16  (0.00-0.41) |
| Overall | 77.53  (66.63-88.44) | 9.55  (6.12-12.99) | 12.73  (6.95-18.50) | 51.42  (31.17-71.67) | 19.65  (11.57-27.73) | 28.85  (14.48-43.51) | 88.10  (82.15-94.04) | 4.25  (2.29-6.21) | 7.46  (3.66-11.26) |
| I^2^ | 98.6% | 94.6% | 98.7% | 99.4% | 97.6% | 99.2% | 97.7% | 92.1% | 98.1% |

**Supplementary Table S5** Prevalence (%) of the early initiation of substance use by country among in-school African adolescent girls.

|  | Cigarette smoking initiation | | | Alcohol use initiation | | | Drug use initiation | | |
| --- | --- | --- | --- | --- | --- | --- | --- | --- | --- |
| **Countries** | Non-initiators | <12 years | ≥12 years | Non-initiators | <12 years | ≥12 years | Non-initiators | <12 years | ≥12 years |
| Benin | 94.47  (91.66-97.27) | 1.77  (0.50-3.04) | 3.75  (1.65-5.86) | 93.54  (89.83-97.26) | 1.08  (0.14-2.02) | 5.36  (1.96-8.75) | 98.11  (96.90-99.32) | 0.68  (0.00-1.37) | 1.20  (0.19-2.20) |
| Liberia | 83.82  (78.23-89.41) | 8.47  (5.32-11.61) | 7.70  (4.63-10.78) | 87.38  (84.43-90.34) | 8.19  (5.71-10.66) | 4.41  (2.73-6.10) | 86.47  (82.08-90.86) | 9.22  (6.04-12.39) | 4.30  (2.44-6.16) |
| Mauritius | 77.31  (71.11-83.51) | 4.68  (3.52-5.84) | 18.00  (12.15-23.85) | 83.24  (81.04-85.43) | 3.58  (1.58-5.58) | 13.17  (10.60-15.73) | 95.64  (93.89-97.39) | 0.86  (0.09-1.62) | 3.49  (2.23-4.75) |
| Mozambique | 95.02  (91.92-98.11) | 3.43  (0.85-6.01) | 1.54  (0-3.18) | 95.72  (93.65-97.78) | 1.80  (0.49-3.10) | 2.47  (1.40-3.54) | 98.20  (97.44-98.95) | 0.92  (0.00-1.97) | 0.87  (0.00-1.81) |
| Namibia | 82.07  (77.61-86.53) | 7.53  (5.50-9.56) | 10.39  (7.31-13.46) | 76.47  (72.89-80.06) | 7.12  (5.58-8.67) | 16.39  (13.52-19.25) | 88.31  (85.87-90.76) | 4.16  (3.10-5.22) | 7.51  (5.15-9.86) |
| Seychelles | 66.49  (62.33-70.64) | 10.79  (8.52-13.06) | 22.71  (18.86-26.56) | 82.40  (78.93-85.87) | 6.39  (4.42-8.35) | 11.20  (8.36-14.04) | 90.01  (87.70-92.31) | 2.88  (1.64-4.11) | 7.10  (5.10-9.11) |
| United Republic of Tanzania | 94.93  (93.32-96.54) | 4.48  (3.09-5.88) | 0.57  (0.16-0.98) | 97.38  (96.22-98.54) | 2.45  (1.34-3.56) | 0.16  (0.00-0.41) | 97.03  (96.01-98.07) | 2.48  (1.56-3.39) | 0.47  (0.03-0.92) |
| Overall | 85.06  (77.62-92.50) | 4.75  (3.62-7.87) | 8.79  (4.40-13.17) | 56.13  (32.96-79.30) | 15.42  (9.27-21.57) | 28.25  (12.30-44.20) | 93.87  (91.40-96.34) | 2.56  (1.31-3.81) | 3.33  (1.68-4.97) |
| I^2^ | 97.1% | 90.3% | 97.2% | 99.5% | 97.9% | 99.3% | 95.0% | 90.7% | 93.7% |
